# Assessment of Physical Activity and Related Factors among Adults with Visual Impairments in Japan

**DOI:** 10.3390/ijerph19042244

**Published:** 2022-02-16

**Authors:** Wakako Tatsuta, Takayo Inayama, Erika Yamanaka, Kazunori Ohkawara

**Affiliations:** 1 College of Sports Sciences, Nihon University, Tokyo 154-8513, Japan; 2 Department of Food and Health Sciences, Faculty of Health and Human Development, The University of Nagano, Nagano 380-8525, Japan; inayama.takayo@u-nagano.ac.jp; 3 Graduate School of Human Health Sciences, Tokyo Metropolitan University, Tokyo 192-0397, Japan; erichi4y@gmail.com; 4 Graduate School of Informatics and Engineering, University of Electro-Communications, Tokyo 182-8585, Japan; ohkawara_kazunori@e-one.uec.ac.jp

**Keywords:** physical activity, health promotion, self-efficacy, blind, visual impairment, accelerometer

## Abstract

In Japan, there is a lack of physical activity data on health and disease among people with visual impairments, making it difficult to develop specific strategies for health promotion. This study aimed to identify moderate-to-vigorous physical activity (MVPA) among people with visual impairments in Japan, to examine the percentage of them who meet the MVPA recommended activity, and to identify factors related to MVPA. In this cross-sectional study, we analyzed a survey of 169 adults with visual impairments. The relationship between MVPA and related factors was analyzed using binomial logistic regression analysis. The median MVPA was 46 min/day for men and 36 min/day for women, respectively, aged < 65 years, and 26 min/day for men and 34 min/day for women, respectively, aged ≥ 65 years. Seventy-eight percent of the subjects achieved the recommendations by the WHO, and 28% of the subjects < 65 years had achieved the MVPA of 60 min/day recommended by the Japanese Active Guide. Self-efficacy (SE) for PA, recommended PA implementation behavior, subjective walking speed, and exercise habits were significantly associated with MVPA. The current status of MVPA time among people with visual impairments in Japan, as revealed by this study, indicates that it is important to set realistic goals and plan a step-by-step process to achieve the recommendation. In the future, it will be important to develop a program that encourages the improvement of SE and promotes PA.

## 1. Introduction

Many studies have been reported on the determinants of health. In some countries, health disparities due to disability have been examined as one of the factors [1,2,3]. This is also true for the visually impaired, who are defined by the WHO as low vision or blind. Previous Australian studies have shown that adults with visual impairments have more than twice the risk of total mortality [1] compared with that of adults who are not visually impaired. It has also been reported that a group with chronic diseases had more visual impairments than the group without [2]. Furthermore, in an analysis of people aged ≥ 65 years from the United States National Health and Nutrition Examination Survey (NHANES), those with visual impairments were more likely to be underweight and had higher biomarkers of lifestyle-related diseases, such as low-density lipoprotein cholesterol and homocysteine, than those without visual impairments [3]. There is an urgent need for efforts to reduce the risk of lifestyle-related disease for people with visual impairments, who are at higher risk of lifestyle-related diseases than those without visual impairments. To achieve this, moderate physical activity (PA) is essential, in addition to a healthy diet and better mental health. In fact, it has been reported that additional PA has a positive effect on body composition and physical fitness in people with visual impairments [4].

Decreased PA is associated with increased obesity, diabetes mellitus, some cancers, and other chronic diseases [5,6,7]. People with visual impairments often limit PA due to fear of falling and perceived risks to mobility [8,9]. In a number of previous international studies, people with visual impairment have also reported low PA. A meta-analysis revealed that children and young adults with visual impairments spent less time on moderate-to-vigorous physical activity (MVPA) than those with normal sight, although the amount of low-intensity activity remained the same [10]. In a study of adult Portuguese men and women [11], the MVPA time was 25 min/day, and in a study of adult Brazilian men and women [12], the percentage of those achieving 30 min/day MVPA was 60%. In a study using NHANES data from the USA, the average number of steps taken per day by people with visual impairments was 5992, about 40% fewer than those taken by people without visual impairments (9964 steps) [13]. Furthermore, MVPA time for people with visual impairments was reported to be 9.3 min/day, 60% shorter than that of people without visual impairments (23.5 min/day) [13]. On the other hand, there is a report that students with visual impairments do not differ from students without visual impairments in PA, such as the number of steps [14], so PA status may differ depending on group attributes.

As a measure to increase PA, PA recommendations and guidelines have been provided by various countries and international organizations. Specifically, the World Health Organization (WHO) recommends that adults should do at least 150–300 minutes of MPA; or at least 75–150 minutes of VPA; or an equivalent combination of MPA and VPA throughout the week, for substantial health benefits. The guideline recommends that for adults living with disability at least 300 min/week of MPA or 150 min/week of VPA is recommended for additional health benefits [15]. The Japanese PA guidelines, the Active Guide [16], recommends 60 min/day of MVPA for those aged 18–64 years, and 40 min/day or more of PA for those aged ≥65 years defined as elderly in Japan, regardless of intensity. As already mentioned, there may be a discrepancy between the guidelines and the current situation in the people with visual impairments [8,9], where PA is more likely to be limited due to the characteristics of the disability. Therefore, it is necessary to understand their actual situation and then plan a practical, step-by-step intervention program to achieve their goals.

Although there are approximately 1.64 million people with visual impairments in Japan, very few studies have clarified the PA of people with visual impairments. Inoue et al. reported in a study using a questionnaire survey that Japanese adults with visual impairments have low PA levels, which may lead to a risk of motor dysfunction [17]. Japanese adults with visual impairments are expected to have lower PA, as in previous international studies. In order to examine stepwise achievement goals based on quantitative data, such as MVPA time and the number of steps taken, it is necessary to conduct research that employs not only questionnaire surveys but also methods that can quantitatively assess daily PA.

Planning an effective PA promotion program for people with visual impairments requires identifying factors associated with MVPA that may promote behavioral change, such as self-efficacy (SE) and environmental factors. In social cognitive theory [18], which describes a dynamic process in which preparatory factors, environmental factors, and behavior mutually influence each other, SE is considered one of the most important factors in promoting behavioral change. Although there are limited studies with people with visual impairments, Haegele et al. reported a positive correlation between self-efficacy and PA (MET-min/wk) in adults with visual impairments [19]. In addition to SE, studies of people without visual impairments have suggested that environmental factors, such as social contact and support by family and friends are related to PA [20,21]. For this population as well, it is necessary to examine whether MVPA is related to preparatory factors, such as SE, and surrounding support.

The purpose of this study was to determine the MVPA time of Japanese adults with visual impairments, who are predicted to have low PA as in previous studies and to examine the percentage of those who meet the recommendation of MVPA (min). The recommendation of MPA and VPA was set at least 150–300 min of MPA or at least 75–150 min of VPA throughout the week in accordance with the WHO recommendation of, or an equivalent combination of MPA and VPA. In addition, The recommendation of MVPA was set at 60 min/day of MVPA for patients aged <65 years, in accordance with the Japanese PA guidelines. Since there is no recommended MVPA for those aged ≥65 years, we set the MVPA to 30 min/day, referring to a previous study [22]. We also aimed to clarify the factors associated with MVPA.

## 2. Materials and Methods

### 2.1. Study Design and Participants

This cross-sectional study was undertaken in Japan between September and December 2019. In total, 184 adults (men: women ratio 120:64) with visual impairments participated in a survey. They all fit the WHO definition of low vision or blind. Participants were recruited in cooperation with the Japan Federation of the Visually Impaired (JFVI). This is because we wanted to recruit participants from all prefectures. The JFVI is the largest organization for people with visual impairments in Japan, with a membership of approximately 50,000 individuals with visual impairments.

The study procedure was as follows. First, the researchers obtained the consent and cooperation of the JFVI, after providing a written and verbal explanation of the study’s significance and methodology. We requested JFVI to prepare people corresponding to the attributes of male and female, adulthood and old age, and JFVA suggested four people as the number of people that can be recruited by member organizations in all prefectures. The JFVI then mailed instructions and application forms to all 61 member organizations in Japan, requesting that each organization recruit four participants. Each member organization explained the study to its members either directly or via email, and permission to disclose contact information was obtained from members who expressed interest in participating. Upon receiving the list compiled by the JFVI, we informed individuals on the list via email or telephone of the survey outline, and accelerometers wrapped in cushioning material were mailed out to the participants. After that, the survey was explained to each participant by telephone. Consent was obtained by verbal confirmation of the participant’s intention to consent and then, with permission, an audio recording of the participant’s name and intention to consent. After obtaining consent, the researcher explained how to wear the triaxial accelerometer and the time frame for wearing it. The researchers explained that data obtained using the triaxial accelerometer would be excluded from the analysis if the wearing time was <10/h day.

The study was conducted in accordance with the tenets of the Declaration of Helsinki and the Ethical Guidelines for Medical and Health Research Involving Human Subjects in Japan. The research protocol was approved by the Institutional Review Board at Nihon University College of Sports Sciences (2017-03).

### 2.2. Attributes and Physical Characteristics

Information concerning participants’ sex, age, and vision levels was obtained from records that had been initially maintained by the JFVI. Information concerning the timing of the disability (congenital or mid-career), the form of residence and employment status, and subjective sense of health were confirmed during the telephone interviews. A participant’s body mass index (BMI) was calculated from self-reported height and weight (weight [kg]/height [m^2^]) data.

### 2.3. Measurements

#### 2.3.1. Physical Activity (PA)

PA was monitored using a verified triaxial accelerometer (Active style PRO HJA-750C, Omron Healthcare Co., Ltd., Kyoto, Japan) [23], which has a sensitivity of 3 mG and a range of ±6 G. The epoch length was 10 s, and an average of absolute values for 10 s was used for the combined acceleration. The wearing time and monitoring period was 7 days, including holidays, between September and November 2019. Holidays were defined as work-free days or weekends. Participants continued to wear it except when they could not (swimming, changing clothes, bathing, etc.) and while sleeping. After the 7-day measurement period, participants mailed the triaxial accelerometer to the researchers. The triaxial accelerometer data were imported into a computer once the device was returned. Data were analyzed only if available for >3 weekdays and >1 weekend day, during which time the device was worn for at least 600 min/day (and removed for < 120 min/day). In this study, we adopted the activity time according to the activity intensity of sedentary behavior (SB; metabolic equivalents [METs], ≤1.5) and MVPA (METs, ≥3.0) from the MET values evaluated every 10 s.

#### 2.3.2. MVPA-Related Factors

The factors associated with MVPA were asked about in a telephone interview. Participants responded to the following questions: (i) “Are you aware of the Active Guide in Japan?” (to determine awareness levels) and (ii) ”Do you know how much PA is recommended for health promotion?” (to determine knowledge levels). Response options were “Yes” or “No”; (iii) ”Are you confident in performing the recommendation of PA?” (to determine SE). Response options were (1) strongly agree, (2) agree, (3) disagree, or (4) strongly disagree; (iv) “Have you implemented the recommendations concerning physical activity levels?”(to determine behavior). Response options were: (1) every day, (2) often, (3) sometimes, (4) on a few occasions, or (5) not at all; (v) ‘Are you in the habit of exercising regularly (defined as ≥30 min once or twice a week or more, for ≥1 year)?” Response options were: ”Yes” or ”No”; (vi) “What is the ambulatory speed compared to the same sex and age group?”. Response options were (1) fast, (2) moderately fast, (3) moderate, (4) moderately slow, or (5) slow; To determine support around them, participants were asked: (vii) “Do you have a friend or someone to exercise with?” Responses were “Yes” or “No.”

### 2.4. Statistical Analysis

Unanswered values were treated as missing values and excluded for each analysis. Data were analyzed at two age classifications: <65 years and ≥ 65 years. The nominal and ordinal variables are expressed as numbers (%). Continuous variables are presented as mean (±SD) or median [1st quartile; 3rd quartile]. MVP, VPA and MVPA time is presented as median [1st quartile; 3rd quartile] because it was non-normally distributed by the Kolmogorov–Smirnov test. In addition, the number of people (%) who achieved the recommendation of PA is shown. MPA and VPA were in accordance with WHO recommendations. MVPA was ≥ 60 min/day for those <65 years, in accordance with the Active Guide. Since the Active Guide recommends at least 40 min/day of PA regardless of intensity without MVPA recommendations for people ≥ 65 years, we used a minimum of 30 min/day of MVPA as the standard, referring to previous research (Chen et al., 2015). Sex and age classification differences were compared using chi-square or Fisher’s exact test for nominal and ordinal variables. Additionally, Continuous variables were compared using the Mann–Whitney U test.

The relationship between MVPA and factors related to MVPA was assessed through univariate and multivariate analyses, respectively, using binomial logistic regression analysis. The dependent variable, MVPA, was dichotomous above or below the median of each age classification. The independent variable was also dichotomous so that the responses from the four or more choices could be approached equally. The multivariate analysis provided two models (Models 1 and 2). Model 1 was adjusted for sex, age classification, area of residence, BMI (standard/other), current employment status, living arrangements, and area of residence. In Model 2, the time of disability and degree of disability were added to the adjustment variables in Model 1.

All statistical analyses were performed using IBM SPSS Statistics 21 (IBM Japan Inc., Tokyo, Japan), and the level of significance was set at 5% for two-tailed tests.

## 3. Results

From 184 participants (men, *n* = 120; women, *n* = 64) who agreed to participate in this study, we excluded those who wore the device for < 10 hours per day, those who had no data for > 4 days including holidays, and those who had a disease that limited exercise (*n* = 15). In total, data were analyzed from 169 participants (men, *n* = 110; women, *n* = 59). Of the 169 participants, those with disabilities other than people with visual impairments were not included.

Table 1 shows the attributes and physical characteristics of the participants. Those who were blind accounted for 68% of all men and 71% of all women. Among the men, 3% had a BMI < 18.5, 28% had a BMI 25–30, and 6% had a BMI ≥ 30. Among the women, 17% had a BMI > 18.5, 14% had a BMI 25–<30, and 3% had a BMI ≥ 30.

Table 2 shows the SB, PA, and percentage of people who achieved the PA recommendation, according to sex and age classification. In the group aged <65 years, the MVPA of men was 46 [28; 67] min/day, and that of women was 36 [26; 54] min/day. In ≥65 years, the MVPA of men was 26 [14; 43] min/day, and that of women was 34 [14; 63] min/day. There was no significant difference by sex in either age group. Recalculation of MPA and VPA per day per week showed that 62 (84%) men and 33 (83%) women <65 years age group and 23 (64%) men and 13 (68%) women ≥65 years and older age group achieved the WHO recommendations. Twenty-three men (31%) and nine women (22%) <65 years of age met the MVPA of 60 min/day. Among those ≥65 years, 16 men (44%) and 12 women (63%) met the MVPA of 30 min/day.

Table 3 shows the response distribution of factors related to MVPA. The items with a high percentage of Yes responses among those < 65 years were ‘high SE for PA’ and ‘no exercise habit’, and the percentage of those with no exercise habit was significantly higher among women than among men. In the age group of ≥ 65 years, the items with the highest percentages of Yes responses were ‘high SE for PA and ‘Performing the recommendation of PA’, and the percentages of those with high SE were significantly higher in women than in men.

Table 4 shows odds ratios for factors related to MVPA. The factors significantly associated with MVPA were SE for PA, recommended PA implementation behavior, ambulatory speed, and exercise habits. However, there was no relationship between MVPA and awareness, knowledge, and the presence of a friend during MVPA.

## 4. Discussion

This study was the first to assess the amount of PA in adults with visual impairments in Japan using a validated instrument [23], and to identify MVPA-related factors. In the participants with visual impairments in this study, many achieved the WHO recommendation, but MVPA time was shorter than the Japanese recommended time and it was not easy to achieve the recommendation of PA. However, even in this population, those with higher MVPA had higher SE for the recommended PA.

The MVPA time of the subjects in this study was not shorter than previous studies of people without visual impairment in other countries [11,12]. However, a previous study of people without visual impairment in Japan indicated mean averages between 76 (±27) minutes/day [24] and 72 (±49) minutes/day [25]. In people with non-visual impairments aged ≥ 65 years, Chen et al. reported a median MVPA of 38 min/day [26], Yasunaga et al. reported a mean MVPA of 50 (±343) min/day [27], and Amagasa et al. reported a mean MVPA of 54 (±40) min/day [28]. In this study, the median is shown because of the non-normal distribution. When recalculation to mean, it is 50 (±30) min/day for men and 43 (±25) min/day for women < 65 years, and 31 (±20) min/day for men and 42 (±29) min/day for women ≥ 65 years. As the data are not from the same survey, a simple comparison cannot be made. Nonetheless, it can be said that the amount of daily PA of adults with visual impairments is lower than that of adults without visual impairments, regardless of sex or life stage.

For those who achieved the WHO recommendation, 78% of all participants. In addition, 31% of men and 22% of women in the group aged <65 years achieved the Japanese Active Guide recommendation 60 min/day of MVPA, while 44% of men and 63% of women in ≥ 65 years achieved 30 min/day of MVPA. Although there are very limited reports on adults without visual impairments in Japan, Kawakami et al. reported that 55% of adults achieved 23 METs/hour/week, which corresponds to 60 min/day of MVPA [29]. The recommended goal according to the Active Guide, MVPA 60 min/day or higher, was shown to require considerable effort for the adults with visual impairments. It has been reported that visual impairments reduce the sense of balance necessary to maintain a high level of PA, increase the fear of falling, and decrease ambulatory speed [12,30]. Nevertheless, it is still important to ensure MVPA, as the appropriate MVPA cut off value to distinguish between frail and non-frail individuals was 43.3 min/day in a Japanese frailty study of community residents aged 65–75 years. The participants in this study had no diseases that would limit exercise, 76% were workers, few had a BMI ≥ 30, and their subjective sense of health was good (Table 1). It is not easy to achieve the recommendation of MVPA even for healthy adults, such as the participants in this study, and it is necessary to gradually increase the dose of MVPA in order to promote health and prevent frailty in old age.

In order to bridge the gap between the actual amount of PA in terms of MVPA time and the recommendation, it is necessary to develop a program that incorporates behavioral science to promote PA. Model 2 included time of disability and degree of disability, but the items associated with MVPA did not change between models 1 and 2. This result might be indicative that the years of impairment had no real effect on the factors measured. In our study, SE, behavior, exercise habits, and ambulatory speed-very fast were found to be the factors associated with PA, as shown in Table 4. In this paper, the focus was on SE, which is considered in social cognitive theory to be one of the most important factors in promoting behavioral change [18]. Many studies in people with and without visual impairments reported a positive association between PA and SE [19,31,32,33]. As in the previous study, SE for PA was positively related to MVPA in this study, while recognition of the Active Guide and knowledge of PA were not. This suggests the importance of focusing on improving SE, rather than providing guides and knowledge, when planning PA promotion programs for this population. Specifically, we can plan a program that uses the small-step technique to set behavioral goals one at a time and accumulate successful experiences, and that uses the modeling technique to share practical methods for promoting PA among participants and enhance SE. In the future, the challenge will be to verify, through intervention studies, the possibility that an improvement in SE will promote behaviors and exercise habits that have been positively associated with PA in this population, resulting in an increase in PA.

Our study had several limitations. First, the participants were not randomly selected but consisted of those who volunteered to participate, and our study may have been biased toward those who were physically active and interested in health promotion. Second, it did not ask about the level of disability according to the WHO definition. Third, many of the members of the organizations targeted in this study are blind, a higher percentage compared with that among people with visual impairments generally in Japan (38% of Japanese people with visual impairments have a Level 1 disability certificate, which is equivalent to being blind); therefore, our findings cannot be generalized. Fourth, only a limited number of items were examined in item relation to MVPA. For example, in particular, in recent years, it has been pointed out that environmental factors, such as support from the surrounding environment and the presence of peers, promote behavioral change in relation to interpersonal factors. In this study, no association was found between MVPA and the presence of friends exercising together, but in the future, the association with other surrounding support and environmental factors, such as the presence of a sports center should be carefully examined. Finally, MVPA may have been underestimated because activity meters were removed during swimming and bathing.

Despite these limitations, the present study clarified the actual status of MVPA among Japanese adults with visual impairments and the percentage of those achieving the recommendation of MVPA. An examination of factors associated with MVPA among people with visual impairments in Japan showed that it is important to focus on improving SE in order to bring about behavioral change. Jian et al. stated that most studies of environmental or behavioral interventions studied for reducing physical activity limitation and preventing falls in older people with visual impairments, the certainty of the evidence is generally low due to poor methodological quality and heterogeneous outcome measurements [34]. Social, psychological, and environmental factors that lead to behavior change based on objective measures of PA in daily living should also be examined.

## 5. Conclusions

The current status of MVPA time among people with visual impairments in Japan, as revealed by this study, indicates that it is important to set realistic goals and plan a step-by-step process to achieve the recommendation. In addition, since SE was identified as a factor associated with MVPA, it is necessary to develop a program with techniques to improve SE in order to promote PA.

## Figures and Tables

**Table 1:** Study participants’ attributes and physical characteristics.

Variables		Age, <65 Years				Age, ≥65 Years			
		Men, *n* = 74		Women, *n* = 40		Men, *n* = 36		Women, *n* = 19	
Blindness, ^1^ *n* (%)		46	(62)	27	(68)	29	(81)	15	(83)
Congenital, ^2^ *n* (%)		40	(54)	19	(48)	13	(36)	6	(32)
Worker, *n* (%)		67	(91)	27	(68)	27	(75)	8	(42)
Living alone, *n* (%)		18	(24)	9	(23)	5	(14)	9	(47)
Height (cm), m (SD)		168.8	(6.6)	154.9	(6.6)	165.4	(6.5)	152.3	(4.9)
Weight (kg), m (SD)		68.5	(12.6)	54.4	(8.0)	64.5	(10.0)	48.3	(6.9)
Body mass index, m (SD)		24.0	(3.9)	22.8	(3.7)	23.5	(3.1)	20.8	(2.7)
BMI, <18.5, *n* (%)		2	(3)	5	(13)	1	(3)	5	(26)
BMI, 18.5–24, *n* (%)		45	(61)	26	(65)	24	(67)	13	(68)
BMI, 25–30, *n* (%)		21	(28)	7	(17)	10	(28)	1	(5)
BMI, >30, *n* (%)		6	(8)	2	(5)	1	(3)	0	(0)
Self-rated health, *n* (%)	Very good	26	(35)	11	(28)	12	(33)	10	(53)
	Good	43	(59)	26	(65)	23	(64)	8	(43)
	Not so good	4	(6)	1	(2)	1	(3)	1	(5)

Missing answers were excluded. ^1^ Blind participants were classified according to a self-report. Others were classified as having low vision. ^2.^ Congenital participants were classified according to a self-report. Abbreviations: BMI, body mass index; *n*, number; m, mean; SD, standard deviation.

**Table 2:** Sedentary behavior and physical activity according to sex and age subgroup classifications.

PA	Age, <65 Years					Age, ≥65 Years					Men	Women
	Men, *n* = 74		Women, *n* = 40			Men, *n* = 36		Women, *n* = 19				
	Median	[1st Quartile; 3rd Quartile]	Median	[1st Quartile; 3rd Quartile]	*p^2^*	Median	[1st Quartile; 3rd Quartile]	Median	[1st Quartile; 3rd Quartile]	*p^2^*	*p^3^*	*p^3^*
Wear time (min/day)	848	[788; 941]	886	[826; 997]	0.106	849	[762; 986]	927	[785; 978]	0.490	0.746	0.924
SB(min/day)	402	[319; 458]	389	[320; 448]	0.693	395	[328; 455]	343	[295; 445]	0.215	0.952	0.313
MPA(min/day)	45	[26, 66]	36	[26, 54]	0.386	26	(14, 43)	34	[15, 57]	0.322	0.002	0.527
VPA (min/day)	0.2	(0, 0.8)	0	[0, 0.3]	0.045	0	[0, 0.2]	0	[0, 0.2]	0.567	0.004	0.913
MVPA(min/day)	46	[28; 67]	36	[26; 54]	0.196	26	[14; 43]	34	[14; 63]	0.184	<0.001	0.901
Ambulatory(min/day)	35	[19; 55]	20	[12; 38]	0.008	17	[8; 32]	30	[7; 38]	0.436	<0.001	0.880
Non-ambulatory (min/day)	9	[5; 16]	15	[8; 22]	0.009	9	[2; 12]	17	[5; 21]	0.034	0.163	0.269
	*n*	(%)	*n*	(%)	*p^4^*	*n*	(%)	*n*	(%)	*p^4^*	*p^5^*	*p^5^*
WHO RecommendedAchievers	62	(84)	33	(83)	0.528	23	(64)	13	(68)	0.489	0.020	0.187
MVPA 60 min/day Achievers	23	(31)	9	(22)	0.387	3	(8)	5	(26)	0.109	0.008	0.749
MVPA 30 min/day Achievers	55	(69)	25	(63)	0.204	16	(44)	12	(63)	0.259	0.003	1.000

Values are number (%) or median [1st quartile; 3rd quartile]. *p2* Compared between the sexes using a Mann-Whitney U test. *p3* Compared between age categories using a Mann-Whitney U test. *p4* Compared between the sexes using a Fisher’s exact test. *p5* Compared between age categories using Fisher’s exact test. Abbreviations: MVPA, moderate-to-vigorous physical activity; n, number; SB, sedentary behavior.

**Table 3:** Related factors for MVPA according to sex and age subgroup classifications.

			Age, <65 Years					Age, ≥65 Years					Men	Women
Related Factors for MVPA	Questions	Alternatives	Men, *n* = 74		Women, *n* = 40			Men, *n* = 36		Women, *n* = 19				
			*n*	(%)	*n*	(%)	*p^3^*	*n*	(%)	*n*	(%)	*p^3^*	*p^4^*	*p^4^*
Awareness ^1^	Are you aware of the “Active Guide”?	Yes	13	(18)	6	(15)	0.798	10	(28)	3	(16)	0.320	0.223	1.000
		No	61	(82)	34	(85)		26	(72)	16	(84)			
Knowledge ^1^	Do you know how much activity time is recommended for health promotion?	Yes	26	(35)	18	(45)	0.320	14	(39)	8	(42)	0.817	0.833	1.000
		No	48	(65)	22	(55)		22	(61)	11	(58)			
Self-efficacy ^1^	Do you feel able to reach the recommended levels with confidence?	Strongly agree	36	(49)	11	(28)	0.089	21	(58)	15	(79)	0.278	0.481	0.001
		Agree	27	(36)	20	(50)		9	(25)	3	(16)			
		Disagree, Strongly disagree	11	(15)	9	(22)		6	(17)	1	(5)			
Behaviour ^1^	Do you implement the recommendations?	Everyday	22	(30)	9	(23)	0.016	16	(44)	10	(35)	0.759	0.336	0.032
		Most of the day	32	(43)	9	(23)		10	(28)	6	(27)			
		Some of the day	14	(19)	12	(30)		8	(22)	2	(13)			
		Not very often, Never	6	(8)	10	(25)		2	(6)	1	(4)			
Exercise habits ^2^	Are you in the habit of exercising?	Yes	33	(45)	8	(20)	0.014	11	(31)	8	(42)	0.392	0.214	0.116
		No	41	(55)	32	(80)		25	(69)	11	(58)			
Ambulatory speed	Is your walking speed as fast, or faster, than those of the same age category and sex?	Fast	35	(47)	10	(25)	0.100	14	(39)	8	(42)	0.838	0.747	0.462
		Moderate fast	17	(23)	10	(25)		11	(31)	5	(26)			
		Moderate	16	(22)	14	(35)		7	(19)	5	(26)			
		Moderate slow, Slow	6	(8)	6	(15)		4	(12)	1	(5)			
Exercising in the presence of friends	Do you have someone to exercise with?	Yes	54	(73)	27	(68)	0.666	16	(44)	17	(90)	0.001	0.094	0.141
		No	20	(27)	13	(32)		20	(56)	2	(11)			

Values are presented as numbers (%). ^1^ The ‘Activity Guide’ refers to the official Japanese activity guidelines for health promotion. This guide recommends 60 min/day of activity of ≥3 METs for people aged 18–64 years and 40 min/day of activity for people aged ≥ 65 years, regardless of intensity, for health promotion. In this study, we asked questions according to each age category. ^2^ Being in the habit of exercising refers to exercise performed for ≥30 min at least twice a week, and continuously for >1 year. *p^3^* Compared between the sexes using a chi-squared test. *p^4^* Compared between age groups according to sex using a chi-squared test. Abbreviations: METs, metabolic equivalents; MVPA, moderate-to-vigorous physical activity.

**Table 4:** Odds ratios and confidence intervals for factors related to MVPA using logistic regression analysis (*n* = 169).

Factors Relating to MVPA	Questions	Univariate Analysis		*p^1^*	Multivariate Analysis					
					Model 1		*p^1^*	Model 2		*p^1^*
		OR (95% CI)			OR (95% CI)			OR (95% CI)		
Awareness of the activity guide	Are you aware of the ‘Active Guide’?	0.83	(0.38–1.81)	0.635	0.83	(0.37–1.85)	0.639	0.74	(0.32–1.71)	0.475
Knowledge	Do you know how much activity is recommended for health promotion?	1.61	(0.86–3.00)	0.136	1.57	(0.83–2.97)	0.164	1.52	(0.79–2.94)	0.209
Self-efficacy	Do you believe you can achieve the recommended level of exercise, with confidence?	3.33	(1.76–6.28)	<0.001	3.66	(1.86–7.20)	<0.001	3.76	(1.87–7.57)	<0.001
Behavior	Have you implemented the recommendations?	3.60	(1.84–7.04)	<0.001	3.90	(1.92–7.91)	<0.001	4.05	(1.94–8.44)	<0.001
Exercise habits	Are you in the habit of exercising regularly?	2.69	(1.41–5.15)	0.003	3.35	(1.67–6.70)	<0.001	3.95	(1.88–8.31)	<0.001
Ambulatory speed, very fast	Are you walking faster than those of the same age category and sex?	2.84	(1.50–5.36)	<0.001	3.42	(1.74–6.73)	<0.001	3.62	(1.79–7.32)	<0.001
Exercise in the presence of friends	Do you have a friend or someone to exercise with?	1.30	(0.68–2.50)	0.427	1.34	(0.68–2.65)	0.396	1.33	(0.67–2.67)	0.214

*p^1^* Correlations of MVPA with each factor were tested using logistic regression analysis adjusted for the following variables: Model 1: sex, age classification, BMI (standard/other), working, living arrangements, area of residence. Model 2: sex, age classification, BMI (standard/other), working, living arrangements, area of residence, time of disability, degree of disability. Abbreviations: BMI, body mass index; CI, confidence interval; MVPA, moderate-to-vigorous physical activity; OR, odds ratio.

## Data Availability

The data presented in this study are available on request from the corresponding author.
